# Nurses’ Cross‐Border Work Intentions Driven by Psychological Empowerment: A Cross‐Sectional Study

**DOI:** 10.1155/jonm/8714790

**Published:** 2026-03-09

**Authors:** Ruoxuan Zhang, Xin Wang, Angela Y. M. Leung, Xiaoyan Jin, Hongxia Dai, Yan Wang, June Zhang, Renli Deng, Angela C. Y. Poon, Ka Wa Mio, Ming Liu

**Affiliations:** ^1^ Faculty of Health Sciences and Sports, Macao Polytechnic University, Macao, China, mpu.edu.mo; ^2^ School of Nursing, The Hong Kong Polytechnic University, Hong Kong, China, polyu.edu.hk; ^3^ Peking University Health Science Center-Macao Polytechnic University Nursing Academy, Macao Polytechnic University, Macao, China, bjmu.edu.cn; ^4^ School of Nursing, Sun Yat-Sen University, Guangzhou, China, sysu.edu.cn; ^5^ Department of Nursing, Zunyi Medical University, Zunyi, China, zmc.edu.cn

**Keywords:** clinical nurses, cross-border work intention, latent profile analysis, psychological empowerment

## Abstract

**Background:**

Given the globalization of the nursing workforce, psychological empowerment represents a critical intrinsic determinant of nurses’ mobility intentions, specifically regarding cross‐border work.

**Aim:**

To identify latent profiles of nurses’ psychological empowerment, examine associated factors, and explore the relationship between these profiles and cross‐border working intention.

**Methods:**

A cross‐sectional multicenter study was conducted from March to September 2023. Using convenience sampling, clinical nurses were recruited through liaisons from nursing societies in nine cities of Guangdong Province. Data were collected through questionnaires covering sociodemographic questionnaire, psychological empowerment, and cross‐border working intention, with analyses including chi‐square tests, logistic regression, and latent profile analysis (LPA) performed using SPSS 23.0 and Mplus 8.3.

**Results:**

A total of 3671 valid questionnaires were collected, and 39.5% of the respondents reported cross‐border intentions. LPA identified three psychological empowerment profiles among nurses, ranked from high to low: the core‐driven empowerment profile (16.94%), the adaptive empowerment profile (70.42%), and the constrained empowerment profile (12.64%). The nurses with lower salary, intermediate title, and without specialist nurse qualification were more likely to fall into the constrained empowerment profile. Psychological empowerment was positively correlated with nurses’ cross‐border work intention. The core‐driven profile showed the highest cross‐border work intention (50.6%), followed by the adaptive (38.2%) and constrained profiles (31.7%). For cross‐border work, the constrained profile prioritized salary (87.1%) as the key concern, while the core‐driven profile focused more on good promotion opportunities (70.3%).

**Conclusion:**

Psychological empowerment exerts a positive impact on clinical nurses’ cross‐border work intention, with the three identified empowerment profiles exhibiting divergent motivational priorities and decision logics. These findings highlight the need for subgroup‐specific strategies to balance nursing workforce mobility and stability.

**Implications for Nursing Management:**

The findings support a differentiated human resource strategy based on nurses’ psychological empowerment profiles. For core‐driven nurses, institutions should provide international career development channels to strengthen their domestic job embeddedness. For adaptive nurses, tailored skill training and decision‐making autonomy should be offered to guide their mobility aspirations. For constrained nurses, competitive compensation and family support services should be prioritized to address their stability needs and rebuild professional confidence. These targeted measures balance talent mobility and domestic workforce stability.

## 1. Introduction

With the acceleration of globalization and the intensification of population aging, cross‐border employment of nurses has become a significant phenomenon in the international labor market. This phenomenon not only embodies the essential international cooperation needed to address global healthcare challenges but also serves as a pivotal mechanism for the integration of global healthcare resources and the development of the nursing profession. In recent years, the cross‐border mobility of nurses from developing countries has provided crucial support for balancing global medical resources. Nurses from India [1] and Southeast Asia [2] are increasingly migrating to Canada, the United States, and the United Kingdom. A survey in certain regions of China revealed that 40.1% of students majoring in international nursing intend to work abroad [3], and the World Health Organization [4] reports that in 2023, high‐salary countries relied on foreign‐trained nurses for 23% of their workforce. Cross‐border employment of nurses, as an important practice in responding to population aging and healthcare resource imbalance in the context of globalization, still holds an irreplaceable core value in promoting international cooperation and global health governance.

However, the driving mechanisms of cross‐border intentions remain underexplored, as existing studies focus more on macro factors than micro psychological ones like psychological empowerment, which has rarely been applied in this field. Therefore, this study aimed to examine the complex interplay of factors influencing nurses’ cross‐border intentions within the framework of psychological empowerment theory.

## 2. Background

Cross‐border employment refers to the practice of individuals engaging in work activities across statutorily defined national borders or administrative boundaries, with their habitual residence or registered permanent domicile as the geographic reference point [5]. Its core criterion lies in whether the employment behavior involves the crossing of formally demarcated borders, rather than whether the employee crosses nationalities. Thus, this concept encompasses both transnational employment across different countries and cross‐regional employment between administratively distinct areas within a single country where systemic institutional divergences exist. Within the context of the Guangdong–Hong Kong–Macao Greater Bay Area (GBA) in China, cross‐regional employment between mainland China, the Hong Kong Special Administrative Region (HKSAR), and the Macao Special Administrative Region (MSAR) is defined as “cross‐border employment,” a well‐established consensus widely recognized in regional academic research, policy practices, and social cognition.

In the specific context of nurses’ cross‐border employment, existing scholarship has primarily framed this decision through the lens of external drivers, including career progression, higher salaries, and diverse clinical experiences [6], while also acknowledging that such cross‐border practice entails navigating considerable challenges like cultural adaptation and professional identity reconstruction [7]. The push–pull theory posits that migration takes place when the expected benefits of a destination outweigh the combined effects of an individual’s current circumstances and migration costs [8]. Smith et al. [9] found that internationalization of healthcare, along with pull factors such as salary gaps, career prospects, and advanced professional experience, drives highly educated nurses’ interest in cross‐border work, a finding corroborated by Wu et al. [10] who emphasized unfair pay as a key push factor prompting nurses to seek overseas positions. For example, the outflow of doctors from Algeria [11] and the choice of Lebanese [12] medical students to pursue advanced studies overseas are both driven by push factors in their home countries, whereas the cross‐border mobility of high‐caliber international talents and scientists is attracted by destination‐side pull factors such as competitive compensation and enabling research environments [13]. Conversely, barriers, including language, professional qualification hurdles, family separation, and difficulty fulfilling family responsibilities (especially for married female nurses), weaken such intentions [14, 15]. Notably, all these identified factors, whether pull or push or barrier factors, are external to individual nurses, leaving the potential role of intrinsic psychological factors largely unexplored in relevant research.

Psychological empowerment is one of the widely used contextual variables in management research, which comprises four cognitive dimensions: meaning, self‐determination, competence, and impact [16]. Studies have shown that psychological empowerment can enhance job satisfaction [17], increase nurses’ engagement in work, improve work quality [18], promote positive behaviors in the workplace [19], and help them better cope with work stress and reduce occupational burnout [20]. We connect the model’s general cognitions to nurses’ experiences of psychological empowerment in managerial work. Meaning refers to the alignment between nurses’ professional values and personal beliefs, highlighting their identification with the value of nursing practice and providing motivational guidance for career choices. Self‐determination is the degree of autonomy nurses perceive in making professional decisions, such as developing care plans and organizing workflows, thereby enhancing their sense of behavioral autonomy [21]. Competence reflects nurses’ self‐efficacy in professional skills, problem‐solving, and adapting to new work environments, providing the necessary ability to achieve their goals [22]. Impact captures nurses’ perception of the effect their work has on patients, teams, and broader healthcare system, reflecting their sense of professional efficacy [23].

**FIGURE 1:**
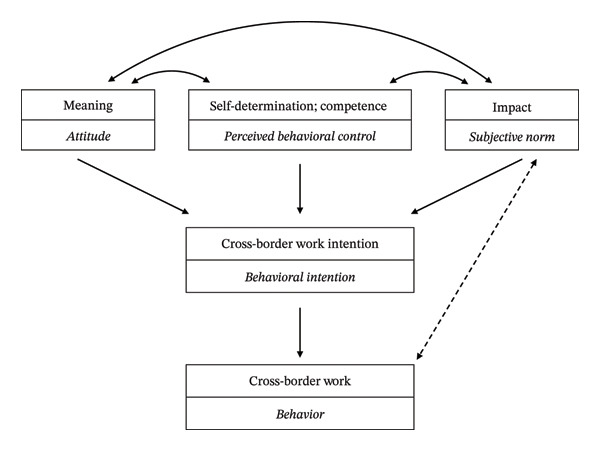
Theoretical framework linking psychological empowerment to cross‐border work intention.

Against this conceptual foundation of psychological empowerment, the theory of planned behavior (TPB) offers a systematic theoretical lens to further interpret the cognitive mechanisms underlying nurses’ cross‐border work intention. TPB was proposed by Ajzen in 1985 as a highly influential theoretical framework in social psychology, designed to explain and predict intentional human behavior [24]. This theory posits that behavioral intention, the most direct predictor of behavior, is jointly influenced by three core constructs: attitude, subjective norm, and perceived behavioral control [25]. The four core dimensions of psychological empowerment represent the concrete manifestation of the three core constructs of TPB in nurses’ occupational cognition and motivation, providing an intrinsic cognitive support for the formation of cross‐border work intention. Specifically, the meaning dimension of psychological empowerment shapes the attitude construct in TPB; the self‐determination and competence dimensions of psychological empowerment align with the perceived behavioral control construct of the theory; and the impact dimension of psychological empowerment strengthens the positive effect of subjective norm. Positive feedback derived from the behavior boosts individuals’ perception of their action effectiveness, thereby enhancing their sense of perceived impact (see Figure 1). Grounded in this correspondence and combined with existing research findings, a strong sense of meaning, which influences the attitude construct, motivates nurses to pursue broader professional value and regard cross‐border work as an extension of their professional mission [26]. As Wang et al. [26] noted, perception of work meaning can significantly enhance job satisfaction and sense of self‐worth, thereby driving nurses to pursue broader career opportunities and development space. Self‐determination, which aligns with the perceived behavioral control construct, fosters nurses’ psychological resilience and relieves anxiety stemming from uncertainties in cross‐border settings [20]. Similarly, competence bolsters nurses’ confidence in meeting cross‐border job requirements and mitigates self‐doubt [27]. Together, these two dimensions elevate nurses’ perceived behavioral control toward cross‐border work [28, 29]. The impact dimension, corresponding to the subjective norm construct, makes nurses who perceive their work impact more willing to expand their professional influence and improve risk tolerance through cross‐border practice, promoting the formation of cross‐border work intention via the guiding role of subjective norms. The synergistic effect of these dimensions amplifies the positive impact on nurses’ intention to work across borders. It may thus be hypothesized that individuals with a strong psychological empowerment could be more inclined to proactively consider cross‐border employment, pending further exploration of the specific nature of the relationship between these two factors. The hypothesized framework is described in Figure 1.

However, given the significant heterogeneity in the psychological states of nurses, traditional clustering methods struggle to accurately capture their underlying subpopulation structures. Latent profile analysis (LPA) is an advanced statistical clustering method based on finite mixture models, whose core purpose is to identify homogeneous subgroups within a heterogeneous population [30]. By analyzing the response patterns of individuals across multiple continuous observed variables, LPA reveals unobserved subpopulation structures and is superior at capturing the complexity that is challenging to interpret using traditional methods. In the field of nursing management, LPA can identify heterogeneous subtypes of nurses with respect to job burnout [31], psychological resilience [32], and other relevant dimensions.

Given these merits and the lack of targeted evidence on the psychological empowerment profiles and cross‐border career decisions of GBA nurses, this study adopts LPA as the core analytical approach to achieve the following research objectives: to investigate the current status of cross‐border work intention among GBA nurses and identify the heterogeneous profiles of psychological empowerment; on this basis, to explore the combined influence mechanism of different psychological empowerment profiles on nurses’ cross‐border work intention, thereby clarifying the internal psychological driving mechanism underlying the formation of cross‐border work intention among nurses in the GBA, and to provide theoretical and empirical support for the coordinated development of regional healthcare.

## 3. Methods

### 3.1. Study Design

A cross‐sectional multicenter study with an exploratory design was conducted. The reporting of this study has been prepared in accordance with the Strengthening the Reporting of Observational Studies in Epidemiology (STROBE) statement guidelines for cross‐sectional studies [33].

### 3.2. Study Participants

This study was conducted in nine cities (Guangzhou, Shenzhen, Zhuhai, Foshan, Huizhou, Dongguan, Zhongshan, Jiangmen, and Zhaoqing) within the Guangdong Province that are officially part of the GBA, which constitutes an ideal setting for investigating nurses’ cross‐border work intention, as the region boasts an advanced economy, concentrated healthcare resources, and frequent cross‐border exchanges. In addition, these cities exhibit a gradient of development levels, ranging from megacities to rapidly developing urban areas, which helps capture internal variations in working conditions, salary structures, and types of healthcare institutions that may influence psychological empowerment and cross‐border intentions.

With reference to the general distribution of nurses in nine cities [34], a convenience sampling method was employed to recruit participants, aiming for a roughly even representation of each city. The sample size was calculated using the following formula:

(1) n=Z1−α/2δ 2p1−p×Deff,

where *α* = 0.05 and *δ* = 5%. The estimated sample rate is *p* = 0.5. The application of the design effect (Deff = 2) was intended to ensure statistical power, and considering a 20% interview attrition rate, the minimum sample size required was calculated as *n* = 960. To ensure robust results in LPA, sufficient sample sizes are essential. While limited data may obscure meaningful class distinctions, larger datasets enhance the reliability of identifying latent profiles by improving statistical power.

Participants’ inclusion criteria were nurses who (a) were registered nurses holding a valid nursing practice certificate from the People’s Republic of China, (b) worked full‐time in the hospital, and (c) voluntarily agreed to participate in the study. Exclusion criteria were as follows: (a) nurses who were not on duty during the study period, including those on vacation, sick leave, personal leave, or continuing education, and (b) among the returned questionnaires, those with missing responses on key variables or showing patterned/identical answers. In practice, a total of 3673 nurses from nine cities in Guangdong Province who held valid nursing practice certificates of the People’s Republic of China were enrolled in this study. Of these, 2 were excluded as invalid due to incomplete data, leaving 3671 samples included in the final analysis.

### 3.3. Measurements

#### 3.3.1. Sociodemographic Questionnaire

The sociodemographic questionnaire, self‐compiled by the researcher based on literature review [5, 9] and expert consultation, covered individual factors (sex, age, education level, and marital status) and work‐related factors (years of work experience, monthly salary, professional title, type of work organization, possession of specialist nurse qualification, and requirement to work night shifts).

#### 3.3.2. Cross‐Border Working Intention

The cross‐border nursing work in this study is operationally defined as nursing practice that includes both transnational employment and cross‐regional employment between the Chinese mainland and the Hong Kong and MSARs within GBA. To measure this construct, we developed a single‐item question: “Would you like to work in nursing across borders?” with answer options: yes, no, and undecided. Multiple‐choice questions assessed factors influencing cross‐border working intention: “What are your primary concerns about taking on cross‐border work?” and “What factors would encourage you to pursue cross‐border employment?”.

#### 3.3.3. Psychological Empowerment Scale

The psychological empowerment scale, developed by Spreitzer [16], was translated into Chinese by Li et al. [35]. The psychological empowerment scale consists of 12 items with a maximum score of 60, including Meaning (1–3 items), Self‐determination (4–6 items), Competence (7–9 items), and Impact (10–12 items). These are five‐point Likert scales, ranging from “*strongly disagree*” (=1) to “*strongly agree*” (=5). The higher the average score, the higher the level of psychological empowerment is. A score of 20 or lower was classified as indicating poor empowerment, while a score exceeding 40 was defined as representing high empowerment [19]. In this study, Cronbach’s *α* coefficient was 0.932.

### 3.4. Data Collection

After obtaining approval from the Research Committee of the Macau Polytechnic University, the nursing societies in nine cities within the GBA were contacted, and liaisons were elected in each city. Informed consent forms and questionnaires were set as QR code–enabled online Questionnaire Star forms and distributed to the liaisons from March to September 2023. The liaisons then conducted surveys in accordance with the inclusion and exclusion criteria, inviting respondents to complete the web‐based questionnaires on mobile devices or personal computers within 10–15 min.

### 3.5. Ethical Considerations

This study was approved by the Ethics Committee of the Macao Polytechnic University (Reference No.: RP/AE‐06/2022/E01). The Helsinki Ethical Guidelines for Human Research were meticulously adhered to throughout the research process. The first part of the survey questionnaire is the “Informed Consent Form” which includes the purpose, methods, and significance of the study. It ensures that participants will not be harmed in any way, that they can decide for themselves whether to participate, and that they have the right to withdraw at any time. All data will be used solely for this study and will not be disclosed to any third party. The research results will be presented in the form of group data in the thesis.

### 3.6. Data Analysis

Statistical analysis was conducted using IBM SPSS 23.0 and Mplus 8.3. For descriptive statistics, means (standard deviation) were used to describe metric data. Frequency (percentages) was used to describe categorical data. To minimize the risk of common method bias resulting from self‐reported questionnaires, Harman’s single‐factor analysis and marker‐variable method were used to assess common method bias.

The LPA was conducted using Mplus 8.3 to examine the latent profiles of psychological empowerment. For the LPA, the mean scores for the dimensions (Meaning, Self‐determination, Competence, and Impact) were used as indicators. Each dimension score was calculated as the standardized average of its three corresponding items, yielding a score ranging from 1 to 5 points. Five LPA models, ranging from the initial (one profile) to the final (five profiles), were estimated by gradually increasing the profile number until the fitness indices had achieved optimal levels. Model fit was assessed using the Akaike information criterion (AIC), Bayesian information criterion (BIC), and sample‐size‐adjusted Bayesian information criterion (aBIC), with smaller values indicating better model fit [36]. The classification effect was evaluated using entropy, which ranges from 0 to 1. An entropy value of ≥ 0.8 indicates high classification accuracy. In addition, the Lo–Mendell–Rubin (LMR) and bootstrap likelihood ratio (BLR) tests were also conducted to calculate the *p* value, with *p* < 0.05 indicating that the current model is a significantly better fit for the data than the former [36]. We also performed LPA models with freely estimated correlations among indicators to account for nonindependence across the psychological empowerment dimensions, in addition to the basic LPA with local independence assumption. Factor mixture models (FMMs) were further fitted for comparative analysis to explore the latent profile structure of psychological empowerment from a more flexible analytical perspective.

The data in this study exhibited a two‐level hierarchical structure, with individual nurses nested within cities. To obtain accurate parameter estimates and valid statistical inferences, we employed a series of two‐level random‐intercept models. A two‐level random‐intercept multinomial logistic regression model was employed to assess the sociodemographic factors associated with the latent profiles of nurses’ psychological empowerment. A two‐level random‐intercept binary logistic regression was used to analyze the association between the latent profiles of nurses’ psychological empowerment and their cross‐border intention. Intraclass correlation coefficients (ICCs) were estimated to quantify the proportion of variance in outcomes attributable to city‐level factors, with all individual‐level predictors (e.g., age, salary) treated as fixed effects.

Pairwise comparisons were conducted using the chi‐square test with Bonferroni correction. The overall desired significance level (*α* = 0.05) was applied. Given that *m* represents the number of independent statistical tests conducted, and there were three tests for pairwise comparisons among the three groups, the new significance level was determined as *α*_adjusted_ = *α*/m = 0.05/3 = 0.017. All statistical tests were two‐sided, and a *p* value less than 0.05 was considered statistically significant.

## 4. Results

### 4.1. Participants’ Characteristics

**FIGURE 2:**
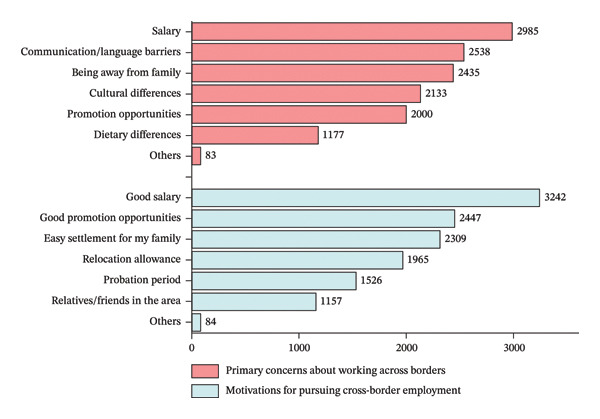
Nurses’ attitudes toward cross‐border work.

Among the 3671 nurses included in this study, females accounted for 94.7%, forming the majority of the sample. The largest age group was those under 30 years old, accounting for the highest proportion of participants (46.4%). In terms of educational background, 45.0% of nurses held a bachelor’s degree or higher, which was lower than the proportion with a college diploma or lower (55.0%). Regarding professional titles, most nurses held junior titles (57.9%), while only 8.8% had senior titles. Among nurses with years of work experience, the highest proportion had 11–20 years (31.3%), and 19.8% had specialist nurse qualifications. In terms of career intentions, 39.5% of nurses expressed a intention to work cross‐border (Table 1). Nurses’ attitudes toward cross‐border work are shown in Figure 2. Primary concern about taking on cross‐border work was “salary” (2985/3671, 81.31%). Among the factors that encourage nurses to pursue cross‐border employment, the primary factor is “good salary” (3242/3671, 88.31%), followed by “good advancement opportunities” (2447/3671, 66.66%).

**TABLE 1:** Sociodemographic characteristics of participants [*n* (%)].

Variables	Overall (*N* = 3671)	Profile 1 (*N* = 464)	Profile 2 (*N* = 2585)	Profile 3 (*N* = 622)	*χ* ^2^	*p*
Sex					1.209	0.546
Male	195 (5.3)	20 (4.3)	143 (5.5)	32 (5.1)		
Female	3476 (94.7)	444 (95.7)	2442 (94.5)	590 (94.9)		
Age					83.515	< 0.001
≤ 30	1704 (46.4)	290 (62.5)	1195 (46.2)	219 (35.2)		
31∼40	1334 (36.3)	131 (28.2)	936 (36.2)	267 (42.9)		
≥ 41	633 (17.2)	43 (9.3)	454 (17.6)	136 (21.9)		
Marital status					34.690	< 0.001
Single	1419 (38.7)	229 (49.4)	992 (38.4)	198 (31.8)		
Married	2252 (61.3)	235 (50.6)	1593 (61.6)	424 (68.2)		
Education					9.693	0.008
Junior college and below	2019 (55.0)	283 (61.0)	1415 (54.7)	321 (51.6)		
Undergraduate education and above	1652 (45.0)	181 (39.0)	1170 (45.3)	301 (48.4)		
Professional title					71.159	< 0.001
Junior title	2125 (57.9)	328 (70.7)	1502 (58.1)	295 (47.4)		
Intermediate title	1222 (33.3)	120 (25.9)	861 (33.3)	241 (38.7)		
Senior title	324 (8.8)	16 (3.4)	222 (8.6)	86 (13.8)		
Years of work experience					95.792	< 0.001
≤ 5	1022 (27.8)	201 (43.3)	693 (26.8)	128 (20.6)		
6∼10	970 (26.4)	120 (25.9)	700 (27.1)	150 (24.1)		
11∼20	1149 (31.3)	104 (22.4)	827 (32.0)	218 (35.0)		
≥ 21	530 (14.4)	39 (8.4)	365 (14.1)	126 (20.3)		
Type of work organization					15.251	0.004
Public hospitals	2934 (79.9)	358 (77.2)	2058 (79.6)	518 (83.3)		
Private hospitals	672 (18.3)	102 (22.0)	482 (18.6)	88 (14.1)		
Others	65 (1.8)	4 (0.9)	45 (1.7)	16 (2.6)		
Monthly salary (CNY)					55.486	< 0.001
< 5000	438 (11.9)	85 (18.3)	301 (11.6)	52 (8.4)		
5000–10000	1997 (54.4)	266 (57.3)	1428 (55.2)	303 (48.7)		
> 10,000	1236 (33.7)	113 (24.4)	856 (33.1)	267 (42.9)		
Whether you need to work night shifts					20.639	< 0.001
Yes	2846 (77.5)	377 (81.3)	2028 (78.5)	441 (70.9)		
No	825 (22.5)	87 (18.8)	557 (21.5)	181 (29.1)		
Whether you have a specialist nurse qualification					34.529	< 0.001
Yes	725 (19.8)	56 (12.1)	505 (19.5)	164 (26.4)		
No	2946 (80.3)	408 (87.9)	2080 (80.5)	458 (73.6)		
Cross‐border work intention					45.960	< 0.001
Yes	1450 (39.5)	147 (31.7)	988 (38.2)	315 (50.6)		
No^a^	2221 (60.5)	317 (68.3)	1597 (61.8)	307 (49.4)		
No (original)^b^	1131 (30.8)	164 (35.3)	794 (30.7)	173 (27.8)		
Undecided (original)^b^	1090 (29.7)	153 (33.0)	803 (31.1)	134 (21.5)		

^a^The combined category of “No” and “Undecided” responses from the original survey, and was used as the comparator group in the dichotomous chi‐square test.

^b^The “No” row represents the original no response option from the survey, while the “Undecided” row represents the original undecided responses, reported separately for transparency.

### 4.2. Common Method Bias Test

We conducted Harman’s single‐factor test on all self‐report measurement items (excluding sociodemographic variables). The first common factor explained only 26% of the total variance (< 50%), suggesting no significant common method bias [37]. Furthermore, we adopted the marker‐variable method, selecting neighborhood relations, a variable theoretically unrelated to the core constructs. After incorporating this variable, the coefficient change rate of the latent profile groups of psychological empowerment on cross‐border work intention ranged from 2.273% to 4.187% (< 10%). Meanwhile, the regression coefficient of the marker variable was statistically nonsignificant (*p* = 0.580), and the model fit showed minimal changes (Table S1). In conclusion, the common method bias in this study was within an acceptable range and would not exert a substantive impact on the research results.

### 4.3. Latent Profile of Nurses’ Psychological Empowerment

In this study, standard LPA was performed on nurses’ psychological empowerment across four dimensions, testing models with 1–5 profiles (see Table 2). The four‐profile model, despite marginally higher entropy (0.928), exhibited insubstantial improvement in information criteria and contained a trivial profile (1.34%) that showed no significant difference in cross‐border intention from low‐empowerment profile, rendering this subdivision practically meaningless and suggesting overfitting. Model comparison indicated that the three‐profile solution demonstrated optimal balance between parsimony and fit (BIC = 23552.495, entropy = 0.918), with distinct and theoretically interpretable profiles characterized by low, medium, and high empowerment levels (12.64%, 70.42%, and 16.94%). However, post hoc diagnostics revealed significant within‐profile correlations (*r* = 0.127–0.512, *p* < 0.01), violating local independence. Sensitivity analyses excluding the highly correlated self‐determination dimension confirmed the robustness of the three‐profile structure (12.16%/71.03%/16.81%), ruling out spurious artifacts (Table S2); nevertheless, given the theoretical centrality of self‐determination and the suboptimal entropy (0.885 < 0.90) upon its exclusion, the full four‐indicator model was retained. Alternative specifications relaxing local independence (LPA with correlated residuals and FMM) yielded severely imbalanced class distributions and entropy values below acceptable thresholds (Table S2), indicating limited practical utility. Therefore, based on statistical adequacy, theoretical coherence, and classification utility, the three‐profile standard LPA with all four indicators was retained as the final model. This solution demonstrated clear gradients in empowerment scores and associated demographic/work characteristics (see Table 1 and Table 3), with high classification reliability (average posterior probabilities: 93.7%, 96.9%, 95.4%; see Table 4).

**TABLE 2:** Fit indices for models with different numbers of latent profile.

Class	*k*	LL	AIC	BIC	aBIC	Entropy	LMR (*p*)	bLRT (*p*)	Proportion (%)
1	8	−15289.240	35761.632	35823.714	35791.939	—	—	—	100
2	13	−13572.946	27171.892	27252.599	27211.291	0.772	< 0.001	< 0.001	27.57/72.43
3	18	−11702.374	23440.747	23552.495	23495.300	0.918	< 0.001	< 0.001	12.64/70.42/16.94
4	23	−10753.645	21553.291	21696.080	21622.997	0.928	0.0003	0.0003	34.32/17.68/1.34/16.67
5	28	−10503.982	21063.964	21237.795	21148.824	0.881	0.0046	0.0051	1.06/56.28/8.42/16.18/18.06

*Note: k*, the free parameters; LL, the loglikelihood.

Abbreviations: aBIC, adjusted Bayesian information criteria; AIC, Akaike information criteria; BIC, Bayesian information criteria; BLRT, bootstrapped likelihood ratio test; LMRT, Lo–Mendell–Rubin test.

**TABLE 3:** Psychological empowerment of participants ($\overset{¯}{\chi }$ ± *s*).

	Overall (*N* = 3671)	Profile 1 (*N* = 464)	Profile 2 (*N* = 2585)	Profile 3 (*N* = 622)	*F*	*p*
Psychological empowerment	45.85 ± 7.13	34.02 ± 5.55	45.40 ± 3.34	56.54 ± 3.39	4989.22	< 0.001
Meaning	12.04 ± 2.03	8.91 ± 2.05	11.99 ± 1.27	14.60 ± 0.81	2439.24	< 0.001
Self‐determination	11.76 ± 1.91	8.62 ± 1.67	11.66 ± 0.98	14.51 ± 0.77	4135.29	< 0.001
Competence	12.03 ± 1.78	9.32 ± 1.91	11.91 ± 0.93	14.54 ± 0.69	3189.99	< 0.001
Impact	10.02 ± 2.59	7.17 ± 2.08	9.84 ± 2.01	12.90 ± 2.31	1052.81	< 0.001

**TABLE 4:** Average belonging probability of samples in each latent profile.

Latent profile	Profile 1 (%)	Profile 2 (%)	Profile 3 (%)
Profile 1	93.7	0.6	0.0
Profile 2	1.7	96.9	0.0
Profile 3	0.0	0.05	95.4

**FIGURE 3:**
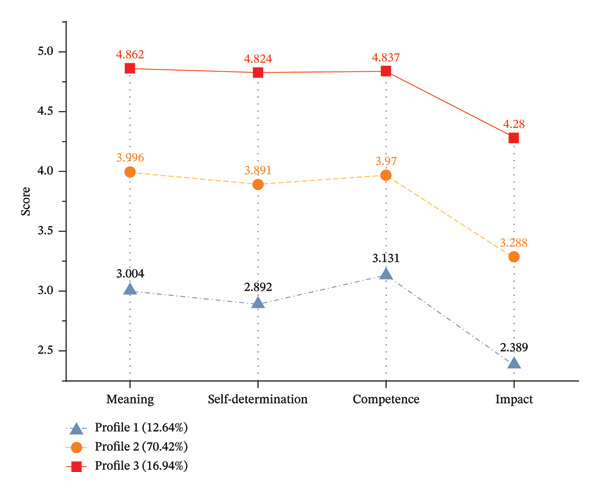
Nurses’ psychological empowerment scores in three profiles.

Based on the results of the LPA, three profiles of psychological empowerment were identified for nurses. The standardized mean scores (Table S3) of the three profiles across the four dimensions of psychological empowerment are shown in Figure 3. The results showed that the total scores and scores of the four dimensions of psychological empowerment were ranked as Profile 1 < Profile 2 < Profile 3, with significant differences for the three profiles of nurses (*p* < 0.05). A total of 464 individuals were included in Profile1, accounting for 12.64%. They had the lowest total scores and scores across all four dimensions of psychological empowerment, earning them the designation of the “Constrained empowerment profile”. Profile 2 comprised 2585 individuals (70.42%), who had medium total scores and scores in all four dimensions of psychological empowerment, and was designated as the “Adaptive empowerment profile.” Profile 3 included 622 individuals (16.94%), who had the highest total scores and scores in all four dimensions, and was designated as the “core‐driven empowerment profile” (see Table 3).

### 4.4. Characteristics Differences Among Three Latent Profiles of Nurses

The results showed significant differences in demographic characteristics among the three latent profiles (*p* < 0.05). Among Profile 3 (high‐empowerment group), the highest proportions were observed for nurses who were 31∼40 years old (42.9%), married (68.2%), with an undergraduate education and above degree (48.4%), holding middle or senior professional titles (38.7% and 13.8%), with more than 10 years of work experience (55.3%), with high salary (42.9%), and working in public hospitals (83.3%). Among Profile 1 (low‐empowerment group), the highest proportions were observed for nurses who were ≤ 30 years old (62.5%), with a junior college education or below (61.0%), holding junior professional titles (70.7%), with ≤ 5 years of work experience (43.3%), with low salary (18.3%), and those required to work night shifts (81.3%) (see Table 1).

### 4.5. Factors Associated With Psychological Empowerment Subgroups

Given the two‐level hierarchical structure of our data, in which individual nurses were nested within cities, a two‐level random‐intercept multinomial logistic regression model was employed in this study, with the core‐driven empowerment subgroup as the reference category, to examine the associations between sociodemographic factors and latent profiles of psychological empowerment among nurses. Random effects analyses revealed that the constrained empowerment subgroup had a random‐intercept variance of 0.081, an ICC of 0.024, and a *Z*‐test value of 1.143 (*p* = 0.253), whereas the adaptive empowerment subgroup had a random‐intercept variance of 0.036, an ICC of 0.011, and a *Z*‐test value of 0.033(*p* = 0.273). Although no significant cross‐group effects were identified in the multilevel analysis, considering the nested structure of the data, we reported the results of the two‐level random‐intercept multinomial logistic regression model, with the findings of the single‐level multinomial logistic regression provided in the appendix (Table S4), and the results of the two analytical approaches were generally consistent.

The fixed‐effects analyses showed significant associations (*p* < 0.05) between psychological empowerment subgroup membership and variables including age, salary, professional title, specialist nurse certification, and night shift. Specifically, compared to the core‐driven empowerment profile, (1) Nurses who had a monthly salary ≤ 5000 CNY (OR = 2.074, 95% CI = 1.292∼3.331), intermediate job title (OR = 2.063, 95% CI = 1.079∼3.945), and without a specialist nurse qualification (OR = 1.733, 95% CI = 1.214∼2.474) were more likely to be in the constrained empowerment profile; (2) Nurses with 11–20 years of work experience (OR = 1.716, 95% CI = 1.088∼2.726), who had a monthly salary ≤ 5000 CNY (OR = 1.446, 95% CI = 1.000∼2.092), and without a specialist nurse qualification (OR = 1.278, 95% CI = 1.026∼1.593) were more likely to be in the adaptive empowerment profile, whereas nurses aged 31–40 (OR = 0.537, 95% CI = 0.340∼0.849) and those who did not need to work night shifts (OR = 0.780, 95% CI = 0.624∼0.976) were less likely to be in the adaptive empowerment profile (Table 5).

**TABLE 5:** Factors associated with latent profiles of nurses’ psychological empowerment (*n* = 3671).

Independent variables	Constrained empowerment profile					Adaptive empowerment profile				
	Estimate	SE	*t*/*Z* ^b^	OR 95% CI	*p*	Estimate	SE	*t*/*Z* ^b^	OR 95% CI	*p*
Fixed Effects^a^										
Gender (ref: female)										
Male	−0.346	0.301	−1.151	0.707 (0.392, 1.276)	0.250	0.010	0.206	0.047	1.010 (0.674, 1.513)	0.963
Age (ref: ≥ 41)										
≤ 30	0.379	0.454	0.835	1.460 (0.600, 3.553)	0.404	−0.327	0.292	−0.119	0.721 (0.407, 1.279)	0.263
31∼40	−0.047	0.383	−0.124	0.954 (0.450,2.020)	0.901	−0.621	0.233	−2.669	0.537 (0.340, 0.848)	0.008
Education level (ref: undergraduate education and above)										
Vocational education and below	−0.019	0.142	0.136	1.020 (0.771, 1.348)	0.892	−0.110	0.102	−1.071	0.896 (0.733, 1.095)	0.284
Marital status (ref: married)										
Single	−0.059	0.169	−0.348	0.943 (0.676, 1.314)	0.728	0.035	0.124	0.284	1.036 (0.812, 1.320)	0.777
Professional title (ref: senior title)										
Junior title	0.538	0.390	1.379	1.712 (0.797, 3.680)	0.168	0.324	0.223	1.451	1.382 (0.893, 2.140)	0.147
Intermediate title	0.724	0.331	2.191	2.063 (1.079, 3.945)	0.029	0.251	0.174	1.442	1.285 (0.914, 1.808)	0.150
Years of work experience (ref: ≥ 21)										
≤ 5	0.737	0.478	1.539	2.090 (0.817, 5.343)	0.124	0.383	0.316	1.211	1.466 (0.789, 2.725)	0.226
6∼10	0.299	0.442	0.675	1.348 (0.566, 3.209)	0.500	0.448	0.279	1.605	1.565 (0.905, 2.705)	0.109
11∼20	0.147	0.390	0.378	1.159 (0.539, 2.490)	0.706	0.540	0.236	2.285	1.716 (1.080, 2.726)	0.022
Monthly salary (ref: ≥ 10,000 CNY)										
≤ 5000	0.730	0.242	3.019	2.074 (1.292, 3.331)	0.003	0.369	0.188	1.960	1.446 (1.000, 2.092)	0.050
5000∼10,000	0.272	0.161	1.689	1.313 (0.957, 1.800)	0.091	0.206	0.109	1.887	1.229 (0.992, 1.523)	0.059
Type of work organization (ref: others)										
Public hospitals	0.698	0.579	1.204	2.009 (0.645, 6.257)	0.229	0.055	0.307	0.179	1.057 (0.579, 1.928)	0.858
Private hospitals	1.106	0.605	1.829	3.023 (0.923, 9.894)	0.067	0.339	0.333	1.018	1.404 (0.730, 2.700)	0.309
Whether you have a specialist nurse qualification (ref: yes)										
No	0.550	0.182	3.027	1.733 (1.214, 2.474)	0.002	0.246	0.112	2.185	1.278 (1.026, 1.593)	0.029
Whether you need to work night shifts (ref: yes)										
No	−0.154	0.166	−0.923	0.858 (0.619, 1.189)	0.356	−0.248	0.114	−2.178	0.780 (0.624, 0.976)	0.029
Random effects										
Random‐intercept variance (*τ* ^2^)	0.081	0.071	1.143	—	0.253	0.036	0.033	1.097	—	0.273
ICC	0.024	0.011								
Model fit indices										
−2 Log likelihood (−2LL)	30114.793									
AICc	30118.796									
BIC	30131.091									

*Note:* ref, reference; AICc, corrected Akaike information criterion.

Abbreviations: BIC, Bayesian information criterion; CI, confidence interval; ICC, intraclass correlation coefficient; OR, odds ratio; SE, standard error.

^a^Core‐driven empowerment profile was used as the reference group.

^b^The *t*/*Z* column reports *t*‐values for fixed effect coefficients and *Z*‐values for the significance test of random intercept variances.

### 4.6. Differences in Cross‐Border Work Intentions Among Nurses in the Three Psychological Empowerment Latent Profiles

The results showed significant differences in cross‐border work intentions among the three latent types of nurses (*p* < 0.05). Pairwise comparisons were conducted using the chi‐square test with Bonferroni correction, with a *p* value less than 0.017 being considered statistically significant. Specifically, cross‐border work intentions were significantly lower in the constrained empowerment profile (Profile 1, 147/464, 31.7%) than in the other two profiles, and lower in the adaptive empowerment profile (Profile 2, 988/2585, 38.2%) than in the core‐driven profile (Profile 3, 315/622, 50.6%), showing a gradient pattern: Profile 1 < Profile 2 < Profile 3 (*p* < 0.017) (Table S5).

Among the three latent profiles, chi‐square test results indicated significant between‐group differences in the distribution of concerns regarding “salary” (*χ* ^2^ = 12.336, *p* = 0.002) and “being away from family” (*χ* ^2^ = 9.147, *p* = 0.010). Specifically, “salary” (87.1%) was most frequently identified as the primary cross‐border concern by the constrained empowerment profile, which also had the highest proportion naming “being away from family” (69.8%) as their issue. In contrast, the core‐driven empowerment profile reported “promotion opportunities” as a primary concern at a significantly higher proportion (57.2%) than the other two groups (*χ* ^2^ = 6.063, *p* = 0.048).

Regarding factors influencing the decision to work cross‐border, nurses in the core‐driven empowerment profile had the highest proportion (70.3%) influenced by “good promotion opportunities”, with a statistically significant difference compared to the other two profiles (*χ* ^2^ = 13.259, *p* = 0.001). Meanwhile, the constrained empowerment profile had the highest proportion (38.1%) influenced by “having relatives or friends in the area,” which also demonstrated statistical significance (*χ* ^2^ = 10.822, *p* = 0.004) (see Figure 4 and Table S6).

FIGURE 4Factors and concerns related to cross‐border intention across three profiles. The radar chart displays the percentage of nurses selecting each concern. (a) Factors of concern when considering cross‐border work; (b) factors influencing the decision to work abroad. Profile 1 (red line): constrained empowerment; Profile 2 (yellow line): adaptive empowerment; Profile 3 (blue line): core‐driven empowerment. ^∗^ *p* < 0.05, ^∗∗^ *p* < 0.01 (Pearson chi‐square test).

**Figure:**
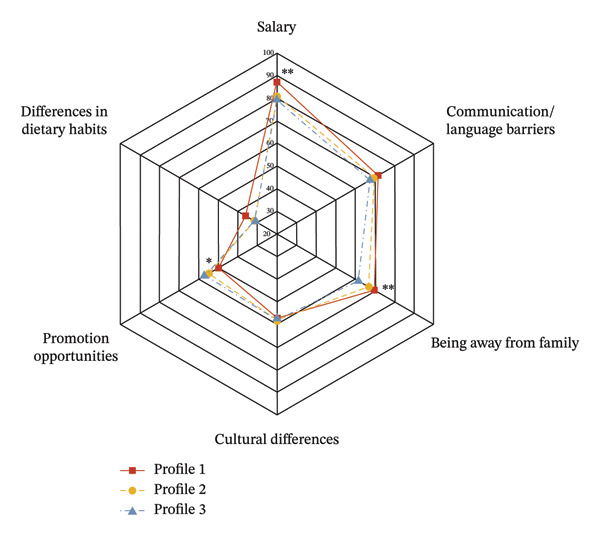
(a)

**Figure:**
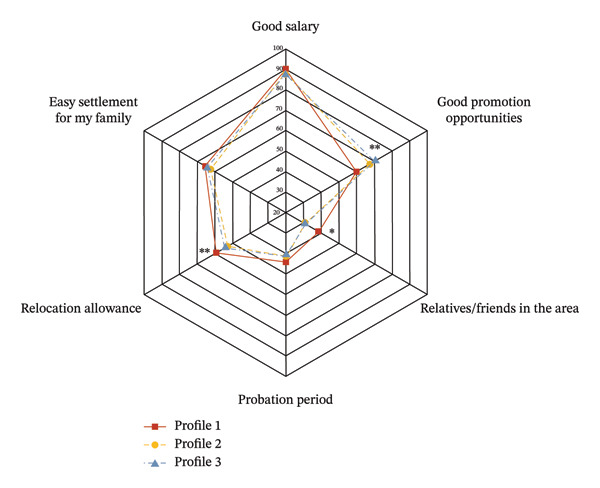
(b)

### 4.7. The Relationship Between Nurses’ Psychological Empowerment Latent Profiles and Cross‐Border Work Intentions

A two‐level random‐intercept logistic regression model was employed to examine the effect of psychological empowerment on cross‐border work intention. ICC revealed that city‐level factors accounted for 0.54% and 0.69% of the variance in cross‐border intention in Model 1 and Model 2, respectively. These results suggest that individual‐level factors are the primary source of variation in cross‐border intention. The results of the binary logistic regression analysis are also provided in the appendix (Table S7), and the findings derived from the two analytical approaches were generally consistent. To focus on the core comparison, we combined the non–cross‐border intention and undecided groups as a unified reference group; sensitivity analyses (Tables S8, S9) verified this grouping was reasonable and the core results were robust, with the undecided group statistically closer to the non–cross‐border intention group.

In Model 1, with the core‐driven empowerment profile as the reference, nurses in the constrained empowerment profile (OR = 0.406, 95% CI = 0.313∼0.528, *p* < 0.001) and the adaptive empowerment profile (OR = 0.572, 95% CI = 0.476∼0.687, *p* < 0.001) demonstrated significantly lower odds of cross‐border intention. Model 2, which used the adaptive empowerment profile as the reference, further confirmed that nurses in the constrained empowerment profile had significantly lower odds of cross‐border intention (OR = 0.706, 95% CI = 0.568∼0.877, *p* = 0.002). These findings suggest that psychological empowerment is positively associated with nurses’ cross‐border intention (Table 6).

**TABLE 6:** Correlation analysis of latent profile and cross‐border work intention.

Independent variables	Estimate	SE	*t*/*Z* ^b^	OR (95% CI)	*p*
Model 1: Core‐driven empowerment profile as reference group					
Fixed Effects^a^					
Constrained empowerment	−0.900	0.134	−6.741	0.406 (0.313, 0.528)	< 0.001
Adaptive empowerment	−0.559	0.093	−5.996	0.572 (0.476, 0.687)	< 0.001
Overall model test (*F*‐test)			*F* = 26.123, df_1_ = 2, df_2_ = 3641		< 0.001
Random effects					
Random‐intercept variance (*τ* ^2^)	0.018	0.015	1.248	—	0.212
ICC	0.0054				
Model fit indices					
−2 Log likelihood (−2LL)	15943.055				
AICc	15945.056				
BIC	15951.255				
Model 2: Adaptive empowerment profile as reference group					
Fixed Effects^a^					
Constrained empowerment	−0.349	0.111	−3.146	0.706 (0.568, 0.877)	0.002
Overall model test (*F*‐test)			*F* = 9.897, df_1_ = 1, df_2_ = 3026		0.002
Random effects					
Random‐intercept variance (*τ* ^2^)	0.023	0.019	1.233	—	0.218
ICC	0.0069				
Model fit indices					
−2 Log likelihood (−2LL)	13277.407				
AICc	13279.408				
BIC	13285.422				

*Note*: Sex, age, education level, marital status, salary, job title, years of work experience, type of work organization, specialist nurse qualification, and working night shifts are covariates; AICc, corrected Akaike information criterion.

Abbreviations: BIC, Bayesian information criterion; CI, confidence interval; ICC, intraclass correlation coefficient; OR, odds ratio; SE, standard error.

^a^Non–cross‐border intention group was used as the reference group.

^b^The *t*/*Z* column reports *t*‐values for fixed effect coefficients and *Z*‐values for the significance test of random intercept variances.

## 5. Discussion

This study conducted an LPA of 3671 clinical nurses’ psychological empowerment in GBA nine cities of Guangdong, China, aiming to explore the relationship between psychological empowerment and cross‐border work intentions to facilitate orderly cross‐border exchange of nursing resources.

### 5.1. Cross‐Border Work Intention of Nurses

These data provide a Chinese case study to support the understanding of the pluralistic model in global nursing talent mobility. This study found that 39.50% of participants intended to work cross‐border, a proportion significantly lower than those in other research. Berşe et al. [38] reported 57% among Turkish nursing students, and Efendi et al. [39] noted a high 91.3% among Indonesian nursing students. A plausible explanation for this discrepancy is that the participants in the present study were predominantly clinical nurses, who had already established mature career paths and signed long‐term employment contracts. In contrast, the samples of the aforementioned two studies were nursing students, whose intention to pursue cross‐border mobility tends to be more flexible. Silvestri et al. [40] demonstrated that nursing students are more inclined than medical students to work abroad, based on research across eight low‐ and middle‐salary countries. This difference is also associated with the specific social and work environments of the participants in the current research. Although the cross‐border work intention among Chinese clinical nurses in the current study was lower than the high proportions documented among nursing student samples in previous international research, this relatively lower intention exactly mirrors the more deliberate decision‐making profile of a mature professional cohort under practical constraints. The intrinsic motivation for such intention has also evolved from the traditional economic‐driven one‐way flow [39] to a pursuit of exposure to international nursing standards and multifaceted career advancement.

### 5.2. The Core‐Driven Empowerment Profile: High Empowerment Drives Proactive Mobility

The core‐driven empowerment profile nurses (16.94%) identified in this study demonstrate a remarkable synergy and reinforcement across the four dimensions of Spreitzer’s [16] psychological empowerment theory. These nurses not only achieved the highest scores across all four cognitive dimensions but also displayed notable demographic characteristics: a predominant age range of 31–40, a high salary, exemption from night shifts, and a high prevalence of specialty certifications. External conditions such as a high salary that recognizes their professional value and exemption from night shifts that preserves their work autonomy work in tandem with internal cognitions, including a strong sense of competence and impact. Together, these factors mutually reinforce one another, forming a self‐sustaining cycle of empowerment. This comprehensive psychological empowerment provides a clear explanation for why this group demonstrated the strongest intention to engage in a cross‐border work (50.6%), with their motivations primarily centered on career development and promotion.

Within the theoretical framework, their profound sense of meaning drives dissatisfaction with established boundaries, prompting them to view cross‐border work as a behavior that expands meaning a way to extend their nursing mission and achieve greater professional value on a broader platform [26, 28]. Secondly, robust self‐efficacy regarding competence forms the foundation of their confidence in evaluating the feasibility of cross‐border movement. They believe their skills are transferable and capable of overcoming challenges like language and licensure requirements, reframing international migration from a daunting risk into a manageable opportunity [27]. Thirdly, habitual self‐determination has shaped their identity as proactive career agents, making the decision to migrate an extension of their active career planning and control, rather than a passive reaction [20]. Finally, their ongoing pursuit of impact leads them to perceive the international stage as an opportunity to gain greater professional impact, participate in cutting‐edge projects, and elevate their professional influence [29]. Their decision‐making framework is rooted in a foundation of strong psychological empowerment. It represents a proactive, growth‐oriented career development strategy, driven by the interplay between internal psychological resources (a four‐dimensional synergy) and external supportive conditions (high salary, senior position). They reframe professional risks as opportunity costs rather than survival threats, and fueled by intrinsic motivation, prioritize how cross‐border practices can facilitate professional breakthroughs. Consequently, during opportunity evaluation, they prioritize developmental resources, such as international research collaboration and leadership cultivation [41], while viewing salary primarily as a reflection of professional value. Efendi’s study [39] on Indonesian nurses similarly found that nursing students motivated by career development or the pursuit of professional experience are more likely to have a clear migration plan compared to those driven by monetary incentives. If domestic healthcare institutions fail to provide career advancement opportunities and development platforms that align with their level of empowerment, such as offering meaningful work, autonomy, and opportunities for impact,these highly capable talents [42], with the greatest potential for leadership and innovation, are most likely to pursue cross‐border mobility to realize their full psychological empowerment.

### 5.3. The Adaptive Empowerment Profile: Moderate Empowerment Triggers Strategic Mobility

As the largest potential mobility group in the sample, the decision‐making mechanism of the adaptive empowerment profile group exhibits characteristics of an equilibrium state. The key challenge for this group is that the four dimensions of psychological empowerment meet baseline levels but fail to create a synergistic effect. While they have the psychological resources needed to perform routine tasks, they lack the internal drive to push beyond the status quo. This leaves them caught between the comfort of their current situation and the perceived potential for growth. The group’s pursuit of a comprehensive compensation and welfare system is essentially a desire to enhance their sense of work meaning through obtaining matched resources and form a positive attitude toward cross‐border intentions. They possess reliable competence and derive some meaning from their work, which supports routine duties [43], but feel less self‐determined and impactful. Instead, their growth may be hindered by structural barriers, such as junior or intermediate professional titles or a lack of specialist nurse qualifications. These factors contribute to career stagnation, limiting their sense of autonomy and influence within their organizations [19].

For this group, cross‐border intent often serves as a backup plan or opportunistic option. Their competence gives them basic confidence to consider cross‐border work, but a lack of autonomy keeps them tied to established routines [19], limiting the proactive, pioneering spirit seen in highly empowered groups. At the same time, their constrained sense of impact weakens their expectation of achieving significant professional growth through cross‐border movement [28]. When it comes to risk perception, they view cross‐border mobility as a cost–benefit calculation. If the perceived costs (leaving a familiar environment, rebuilding professional identity) and potential benefits (career development, better pay) seem evenly balanced, they often hesitate to make decisions [7]. This hesitation reflects their reliance on external situational cues rather than intrinsic motivation. The subjective norm construct in the TPB also influences the behavioral intentions of this group. For example, observing the successful cross‐border mobility of peers is essentially a cue from external subjective norms, which may trigger their intention to work abroad. However, such intentions driven by external norms often lack the support of internal attitudes and perceived behavioral control, making it difficult to translate into actual behavior.

Laari et al. [44] found that Ghanaian nursing students primarily plan to work abroad due to low wages and poor conditions. However, nurses in GBA focus on salary and promotion opportunities reflect dissatisfaction with unmet empowerment needs, but their intrinsic drive is insufficient to support bold or pioneering decisions [17]. To disrupt this state of indecision, managerial interventions should focus on enhancing psychological empowerment within the domestic workplace. For example, expanding clinical autonomy or implementing contribution visualization systems could help them reevaluate the benefits of staying versus leaving [9]. Job resources, when aligned with their needs, can significantly boost intrinsic motivation and well‐being [45]. For the adaptive empowerment group, interventions must go beyond generic incentives. Instead, they should address the gap between competence and opportunity, carefully analyzing whether their behaviors are driven by controlled or autonomous motivation. This targeted approach can help design more effective empowerment and support strategies.

### 5.4. The Constrained Empowerment Profile: Systemic Deficits Motivate Compensatory Mobility

The constrained empowerment profile is marked by pragmatic and risk‐averse characteristics. Its dimensions of psychological empowerment are consistently at low‐to‐mid levels, with significant deficiencies in impact, leading to a state of comprehensively constrained empowerment. Demographically, this group is strongly associated with low salary, lack of specialty certification, and lower professional titles. These structural factors contribute directly to their empowerment deficit: low salary reduces work to a means of survival, eroding meaning; lack of certification and low titles limit autonomy and impact; and prolonged exposure to such conditions further undermines confidence in their competence [19]. These features create a self‐reinforcing loop, constraining their career expectations and developmental agency. This lack of psychological empowerment is directly tied to their low intention for cross‐border work, with motivations heavily driven by extrinsic compensation and survival needs. A significant proportion cited salary as their primary concern, reflecting a survival‐oriented mindset where work is mostly seen as a source of salary [10]. This aligns with Berşe et al. [38], who observed that economic factors are a dominant driver of migration among nursing students with low‐to‐middle salaries. In addition, their high concern about being away from family and reliance on having relatives or friends at the destination highlight their strong risk aversion and dependence on external support networks. These factors stem from their low autonomy and competence, preventing them from adopting the confident, risk‐taking mindset seen in the highly empowered.

Theoretically, this group’s interest in cross‐border work is not driven by aspirations for meaning or professional growth but by compensatory motives [27]. They view geographical mobility as a way to address their lack of economic security and professional dignity (low meaning) in their current roles. Their goal is to find environments with stronger external support, such as family or friend networks, to compensate for their internal deficits in autonomy and competence [15]. In terms of risk perception, they see cross‐border work as a loss of stability rather than an opportunity for growth, internalizing fears about being away from family as a deeper anxiety about losing their core support system [14]. As a result, their cross‐border intention is less an active career strategy and more a passive, survival‐driven decision [2]. This pattern aligns with Ghimire’s [46] findings on nursing students in Nepal, where institutional factors such as inefficient healthcare systems and limited career opportunities drive migration. It also highlights how differences in healthcare systems and economic development influence migration patterns across countries, with varying intensity and motivations.

From a management perspective, an intervention framework combining risk mitigation and capacity building is essential. On the one hand, providing clear cross‐border support pathways, cross‐cultural adaptation training, and peer support systems can reduce their risk perception [7]. On the other hand, substantial improvements in the domestic work environment are critical. These include enhancing pay equity, providing capacity‐building opportunities, offering psychological support, and fostering a respectful and inclusive team culture. Systematic skills training, staged empowerment, and mechanisms for recognizing achievements can gradually rebuild their professional competence and autonomy [45]. By addressing these structural and psychological barriers, organizations can reduce the likelihood of passive, survival‐driven mobility and support the reconstruction of their psychological empowerment.

## 6. Implications for Nursing Management

This study highlights how the four dimensions of psychological empowerment shape differences in nurses’ cross‐border intentions through their interactions, providing a strong theoretical basis for stratified human resource management [47]. Core‐driven empowerment nurses are highly empowered, with proactive mobility driven by career‐oriented motivations. To retain these professionals, healthcare institutions should offer structured international development opportunities aligned with their growth and intrinsic motivations. Strategies include competency‐based promotion systems, short‐term international clinical rotations, leadership roles in cross‐border quality improvement projects, and funding for global conferences [46]. These initiatives directly address their needs for career development and recognition, redirecting their mobility aspirations toward contributing to organizational growth. Adaptive empowerment nurses form the largest group, characterized by moderate empowerment and strategic mobility. To strengthen their domestic engagement, interventions should focus on enhancing their competence and autonomy. Tailored skill development programs, clear specialization pathways, and greater involvement in unit‐level decision‐making [41] can build their confidence and expand their professional scope. These strategies can effectively shift their mobility intentions toward internal career development. Constrained empowerment nurses have low scores across all dimensions of psychological empowerment, with systemic deficits that drive compensatory mobility. For this group, management should focus on stability and foundational support to address their financial and family‐related concerns. Measures such as competitive compensation packages, family support services [48], and comprehensive onboarding programs for cross‐border roles can help reduce their perceived risks [49]. Domestically, gradually increasing task autonomy, offering peer mentorship, and emphasizing the value of nurses’ professional roles can rebuild their sense of competence and self‐worth.

By aligning management strategies with these psychological profiles, healthcare authorities can achieve two key objectives: creating structured international mobility pathways for career‐driven nurses while ensuring a stable and engaged domestic workforce [50]. This differentiated, evidence‐based approach optimizes the allocation of nursing talent within the healthcare system.

## 7. Conclusion

This study identified three distinct latent profiles based on psychological empowerment among clinical nurses: the core‐driven empowerment profile (highest empowerment), the adaptive empowerment profile (moderate empowerment), and the constrained empowerment profile (lowest empowerment). Nurses in the core‐driven profile demonstrated the strongest cross‐border work intention. The profiles were significantly associated with demographic and work‐related factors, such as age, salary, and professional experience. By classifying subgroups with differing mobility intentions based on their psychological empowerment profiles, orderly mobility mechanisms can be established for nurses suitable for cross‐border practice, while empowerment enhancement and retention support can be strengthened for key groups critical to workforce stability, thereby balancing the efficiency of talent mobility and the stability of the regional nursing workforce.

## 8. Strengths and Limitations

Our study has several notable strengths. Primarily set against GBA’s unique political and geographical environment, this research is the first to explore cross‐border work intentions of GBA nurses, while the selection of geographically representative regions enhances the external validity and generalizability of the study findings. Second, the large sample size ensures sufficient statistical power to detect meaningful relationships. Taking psychological empowerment as the pivotal driver, it employs LPA to identify heterogeneous subgroups classified by psychological empowerment and systematically analyzes their relationships with cross‐border work intentions, providing detailed implications for developing targeted strategies regarding nursing mobility.

Despite these strengths, this study has several limitations. First, it was cross‐sectional; it cannot establish causal relationships among the variables. Second, the use of a convenience sampling method limits the generalizability of our findings. Although convenience sampling was adopted, we took the approximate distribution of nurses across the nine GBA cities into account during recruitment to ensure relatively balanced representation across regions, and the large sample size combined with a two‐level model supports the robustness of the LPA. However, unobserved selection bias may still exist. As a result, while the identified psychological empowerment profiles and their associations provide valuable insights into the internal heterogeneity of the sampled cohort, caution is warranted when extrapolating the results to the broader population of clinical nurses in China. The results are most applicable to contexts similar to our study setting. Third, although the test results indicated no common method bias, relying on a single item to measure cross‐border work intention and using self‐reported data collection may compromise the comprehensiveness and reliability of construct measurement. This could also introduce risks of social desirability and recall bias.

Future research can build on the current findings to further explore the mechanisms influencing nurses’ cross‐border work intentions, optimize organizational support and work environments to enhance psychological empowerment, and gain a deeper understanding of the real experiences and needs of nurses working cross‐border. This will provide targeted policy recommendations.

## Author Contributions

Ming Liu: conceptualization, methodology, project administration, supervision, and writing–review and editing. Ruoxuan Zhang: data analysis and writing–original draft. Xin Wang: methodology, data analysis, and writing–review and editing. Angela Y. M. Leung: conceptualization, project administration, and writing–review and editing. Xiaoyan Jin: data collection and writing–review and editing. Hongxia Dai: data collection and writing–review and editing. Yan Wang: data collection and writing–review and editing. June Zhang: conceptualization, project administration, and writing–review and editing. Renli Deng: conceptualization, project administration, and writing–review and Editing. Angela Y. M. Leung: data collection and writing–review and editing. Ka Wa Mio: data collection and writing–review and editing.

There is a statistician on the author team: Professor Ming Liu, Email: karryliu@mpu.edu.mo.

## Funding

This work was supported by the Academic Research Funding of Macao Polytechnic University (grant number: RP/AE‐06/2022).

## Ethics Statement

All procedures for the design and conduct of this study adhered strictly to the Declaration of Helsinki. This study received approval from the Institutional Review Board of the Academic Committee at Macao Polytechnic University (No. RP/AE‐06/2022). All participants completed electronic written informed consent in the integrated module of the QR‐coded online questionnaire before proceeding with data collection.

## Conflicts of Interest

The authors declare no conflicts of interest.

## Supporting Information

Additional supporting information can be found online in the Supporting Information section (Supporting Information).

## Supporting information


**Supporting Information 1** TABLE S1: Common method bias test using the measured marker variable approach.

**Supporting Information 2** TABLE S2: Fit indices for models with different numbers of latent profiles.

**Supporting Information 3** TABLE S3: Latent profile characteristics of psychological empowerment.

**Supporting Information 4** TABLE S4: Multinomial logistic regression analysis of latent profiles.

**Supporting Information 5** TABLE S5: Cross‐border intention among latent empowerment profiles.

**Supporting Information 6** TABLE S6: Factors and concerns related to cross‐border work intention across latent empowerment profiles.

**Supporting Information 7** TABLE S7: Binary logistic regression of cross‐border intention.

**Supporting Information 8** TABLE S8: Cross‐border intention (yes, no, undecided) across latent empowerment profiles.

**Supporting Information 9** TABLE S9: Cross‐border intention by latent empowerment profiles (undecided group excluded).

## Data Availability

The data that support the findings of this study are available from the corresponding author upon reasonable request.
