# Supplementary material for: Nurses’ Cross‐Border Work Intentions Driven by Psychological Empowerment: A Cross‐Sectional Study
Source: J Nurs Manag. 2026 Mar 9;2026:8714790. doi: 10.1155/jonm/8714790 (PMC12968889; doi:10.1155/jonm/8714790)
Supplement: Supplementary file 1 — Supporting Information 1 TABLE S1: Common method bias test using the measured marker variable approach. [file JONM-2026-8714790-s007.docx]

TABLE S1 Common method bias test using the measured marker variable approach

| Model Type | Key Independent Variables | OR (95% CI) | *P* | Coefficient Change (%) | Model Fit Index (-2Log L) |
| --- | --- | --- | --- | --- | --- |
| Model A (Without marker variable) | Constrained empowerment group | 0.406 (0.313, 0.528) | <0.001 | - | 15943.055 |
|  | Adaptive empowerment group | 0.572 (0.476, 0.687) | <0.001 | - |  |
| Model B (With marker variable) | Constrained empowerment group | 0.423(0.314,0.569) | <0.001 | 4.187 | 15947.844 |
|  | Adaptive empowerment group | 0.585(0.479,0.714) | <0.001 | 2.273 |  |
|  | Marker variable: Neighborhood Relations | 1.027(0.934,1.129) | 0.580 | - |  |
| Notes:  The marker variable adopts the total score of the " Neighborhood Relations”, which has no direct theoretical association with psychological empowerment or cross-border work intention and was not included in the main research model.  Core-Driven empowerment profile as reference group.  OR (Odds Ratio); 95% CI (95% Confidence Interval).  Calculation formula for coefficient change: \|(Model B OR - Model A OR) / Model A OR\| × 100%.  Controlled variables: Age, gender, educational level, monthly salary, years of work experience, professional title, night shift status, and specialist nurse qualification (consistent with the main model). | | | | | |
