# Supplementary material for: Nurses’ Cross‐Border Work Intentions Driven by Psychological Empowerment: A Cross‐Sectional Study
Source: J Nurs Manag. 2026 Mar 9;2026:8714790. doi: 10.1155/jonm/8714790 (PMC12968889; doi:10.1155/jonm/8714790)
Supplement: Supplementary file 2 — Supporting Information 2 TABLE S2: Fit indices for models with different numbers of latent profiles. [file JONM-2026-8714790-s008.docx]

TABLE S2 Fit indices for models with different numbers of latent profile

| Model | Profile | k | LL | AIC | BIC | aBIC | Entropy | LMR(*P*) | bLRT(*P*) | Proportion （%） |
| --- | --- | --- | --- | --- | --- | --- | --- | --- | --- | --- |
| LPA | 1 | 8 | -15289.240 | 35761.632 | 35823.714 | 35791.939 | - | - | - | 100 |
| LPA | 2 | 13 | -13572.946 | 27171.892 | 27252.599 | 27211.291 | 0.772 | <0.001 | <0.001 | 27.57/72.43 |
| LPA | **3** | **18** | **-11702.374** | **23440.747** | **23552.495** | **23495.300** | **0.918** | **<0.001** | **<0.001** | **12.64/70.42/16.94** |
| LPA | 4 | 23 | -10753.645 | 21553.291 | 21696.080 | 21622.997 | 0.928 | 0.0003 | 0.0003 | 34.32/17.68/1.34/16.67 |
| LPA | 5 | 28 | -10503.982 | 21063.964 | 21237.795 | 21148.824 | 0.881 | 0.0046 | 0.0051 | 1.06/56.28/8.42/16.18/18.06 |
| LPA(-S) | 2 | 2 | -10809.280 | 21638.561 | 21700.643 | 21668.868 | 0.727 | <0.001 | <0.001 | 25.33/74.67 |
| LPA(-S) | 3 | 3 | -9815.707 | 19659.413 | 19746.328 | 19701.843 | 0.885 | <0.001 | <0.001 | 12.16/71.03/16.81 |
| LPA(-S) | 4 | 4 | -9141.224 | 18318.447 | 18430.195 | 18373.000 | 0.955 | <0.001 | <0.001 | 21.14/16.07/2.18/60.62 |
| LPAc | 2 | 15 | -12286.088 | 24602.175 | 24695.299 | 24647.636 | 0.765 | 0.0001 | 0.0002 | 20.35/79.65 |
| LPAc | 3 | 20 | -11179.198 | 22398.397 | 22522.561 | 22459.011 | 0.894 | <0.001 | <0.001 | 70.91/11.19/17.90 |
| FMM | 2 | 14 | -10858.457 | 21744.914 | 21831.829 | 21787.344 | 0.987 | <0.001 | <0.001 | 1.12/98.88 |
| FMM | 3 | 16 | -10813.993 | 21659.985 | 21759.317 | 21708.477 | 0.835 | 0.0486 | <0.001 | 14.33/1.72/83.96 |
|  | | | | | | | | | | |

*Notes:* LPA = standard LPA with four indicators; LPA(-S) = sensitivity analysis excluding Self-determination dimension; LPAc = LPA with correlated residuals; FMM, Factor Mixture Model.

Abbreviations: k, the free parameters; LL, the loglikelihood; AIC, akaike information criterion; BIC, bayesian information criteria; aBIC, adjusted bayesian information criteria; LMRT, Lo–Mendell–Rubin Test; BLRT, Bootstrapped Likelihood Ratio Test.
