# Supplementary material for: Nurses’ Cross‐Border Work Intentions Driven by Psychological Empowerment: A Cross‐Sectional Study
Source: J Nurs Manag. 2026 Mar 9;2026:8714790. doi: 10.1155/jonm/8714790 (PMC12968889; doi:10.1155/jonm/8714790)
Supplement: Supplementary file 3 — Supporting Information 3 TABLE S3: Latent profile characteristics of psychological empowerment. [file JONM-2026-8714790-s001.docx]

TABLE S3 Latent profile characteristics of psychological empowerment [Mean (SE)]

| **Indicators** | Profile 1  (N=464) | Profile 2  (N=2585) | Profile 3  (N=622) |
| --- | --- | --- | --- |
| Meaning | 3.004 (0.074) | 3.996 (0.014) | 4.862 (0.012) |
| Self-determination | 2.892 (0.056) | 3.891 (0.014) | 4.824 (0.016) |
| Competence | 3.131 (0.063) | 3.970 (0.011) | 4.837 (0.012) |
| Impact | 2.389 (0.040) | 3.288 (0.020) | 4.280 (0.045) |
