# Supplementary material for: Nurses’ Cross‐Border Work Intentions Driven by Psychological Empowerment: A Cross‐Sectional Study
Source: J Nurs Manag. 2026 Mar 9;2026:8714790. doi: 10.1155/jonm/8714790 (PMC12968889; doi:10.1155/jonm/8714790)
Supplement: Supplementary file 4 — Supporting Information 4 TABLE S4: Multinomial logistic regression analysis of latent profiles. [file JONM-2026-8714790-s002.docx]

TABLE S4 Multinomial logistic regression analysis of latent profiles (*n* = 3,671)

| Independent variables | Constrained Empowerment Profile | | | | | Adaptive Empowerment Profile | | | | |
| --- | --- | --- | --- | --- | --- | --- | --- | --- | --- | --- |
|  | B | SE | Wald | OR 95% CI | *P* | B | SE | Wald | OR 95% CI | *P* |
| **Gender (ref: Female)** |  |  |  |  |  |  |  |  |  |  |
| Male | -0.270 | 0.299 | 0.814 | 0.763(0.425,1.373) | 0.309 | 0.050 | 0.205 | 0.059 | 1.051(0.703,1.572) | 0.807 |
| **Age (ref: ≥41)** |  |  |  |  |  |  |  |  |  |  |
| ≤30 | 0.390 | 0.454 | 0.738 | 1.477(0.606,3.187) | 0.430 | -0.321 | 0.292 | 1.207 | 0.726(0.410,1.286) | 0.272 |
| 31~40 | -0.030 | 0.384 | 0.006 | 0.971(0.458,20059) | 0.854 | -0.618 | 0.232 | 7.070 | **0.539(0.342,0.850)** | **0.008** |
| **Education level (ref: Undergraduate education and above)** | | | |  |  |  |  |  |  |  |
| Vocational education and below | -0.012 | 0.140 | 0.007 | 0.988(0.751,1.300) | 0.932 | -0.101 | 0.101 | 1.002 | 0.904(0.742,1.010) | 0.317 |
| **Marital status (ref: Married)** |  |  |  |  |  |  |  |  |  |  |
| Single | -0.052 | 0.169 | 0.095 | 0.949(0.682,1.321) | 0.647 | 0.030 | 0.123 | 0.061 | 1.031(0.810,1.313) | 0.805 |
| **Professional title (ref: Senior title)** |  |  |  |  |  |  |  |  |  |  |
| Junior title | 0.572 | 0.388 | 2.170 | 1.771(0.828,3.789) | 0.141 | 0.333 | 0.221 | 2.274 | 1.396(0.905,2.152) | 0.132 |
| Intermediate title | 0.729 | 0.329 | 4.900 | **2.073(1.807,3.925)** | **0.026** | 0.244 | 0.173 | 1.996 | 1.158(0.910,1.790) | 0.158 |
| **Years of work experience (ref: ≥21)** |  |  |  |  |  |  |  |  |  |  |
| ≤5 | 0.696 | 0.478 | 2.121 | 2.006(0.786,5.121) | 0.145 | 0.352 | 0.314 | 1.255 | 1.422(0.768,2.634) | 0.263 |
| 6~10 | 0.285 | 0.443 | 0.413 | 1.329(0.558,3.166) | 0.520 | 0.443 | 0.278 | 2.529 | 1.557(0.902,2.687) | 0.112 |
| 11~20 | 0.139 | 0.391 | 0.126 | 1.149(0.534,2.472) | 0.723 | 0.544 | 0.236 | 5.312 | **1.722(1.085,2.734)** | **0.021** |
| **Monthly salary (ref: ≥10,000 CNY)** |  |  |  |  |  |  |  |  |  |  |
| ≤5,000 | 0.700 | 0.234 | 8.948 | **2.014(1.273,3.187)** | **0.003** | 0.401 | 0.183 | 4.795 | **1.493(1.043,2.138)** | **0.029** |
| 5,000~10,000 | 0.240 | 0.158 | 2.325 | 1.272(0.934,1.732) | 0.127 | 0.210 | 0.107 | 3.868 | **1.234(1.001,1.521)** | **0.049** |
| **Type of work organization (ref: others)** |  |  |  |  |  |  |  |  |  |  |
| Public hospital | 0.676 | 0.576 | 1.378 | 1.966(0.636,6.073) | 0.240 | 0.093 | 0.303 | 0.094 | 1.098(0.606,1.988) | 0.759 |
| Private hospitals | 0.995 | 0.592 | 2.826 | 2.705(0.848,8.631) | 0.093 | 0.337 | 0.322 | 1.091 | 1.400(0.744,2.633) | 0.296 |
| **Whether you have a specialist nurse qualification (ref: Yes)** | | | |  |  |  |  |  |  |  |
| No | 0.526 | 0.181 | 8.481 | **1.692(1.187,2.410)** | **0.004** | 0.230 | 0.112 | 4.227 | **1.258(1.011,1.567)** | **0.040** |
| **Whether you need to work night shifts (ref: Yes)** | | | |  |  |  |  |  |  |  |
| No | -0.177 | 0.163 | 1.169 | 0.838(0.609,1.155) | 0.280 | -0.268 | 0.112 | 5.717 | **0.765(0.614,0.953)** | **0.017** |
| Note: Core-Driven empowerment profile was used as the reference group.  Abbreviations: ref, reference; SE, standard error; OR, odds ratio; CI, confidence interval. | | | | | | | | | | |
