# Supplementary material for: Nurses’ Cross‐Border Work Intentions Driven by Psychological Empowerment: A Cross‐Sectional Study
Source: J Nurs Manag. 2026 Mar 9;2026:8714790. doi: 10.1155/jonm/8714790 (PMC12968889; doi:10.1155/jonm/8714790)
Supplement: Supplementary file 5 — Supporting Information 5 TABLE S5: Cross‐border intention among latent empowerment profiles. [file JONM-2026-8714790-s003.docx]

TABLE S5 Cross-border intention among latent empowerment profiles

| Comparison Pair | χ² | Crude P-value | Significance |
| --- | --- | --- | --- |
| Profile1 vs Profile2 | 7.199 | 0.007 | **P*<0.017 |
| Profile1 vs Profile3 | 39.091 | ＜0.001 | **P*<0.017 |
| Profile2 vs Profile3 | 32.075 | ＜0.001 | **P*<0.017 |
| Overall | 45.960 | ＜0.001 | **P*<0.05 |

Note: Pairwise comparisons were conducted using the Pearson chi-square test with Bonferroni correction to control for type I error. The adjusted significance level was set at α' = 0.05 / 3 ≈ 0.017.

* indicates a statistically significant difference at the corrected level of α' = 0.017.
