# Supplementary material for: Nurses’ Cross‐Border Work Intentions Driven by Psychological Empowerment: A Cross‐Sectional Study
Source: J Nurs Manag. 2026 Mar 9;2026:8714790. doi: 10.1155/jonm/8714790 (PMC12968889; doi:10.1155/jonm/8714790)
Supplement: Supplementary file 6 — Supporting Information 6 TABLE S6: Factors and concerns related to cross‐border work intention across latent empowerment profiles. [file JONM-2026-8714790-s009.docx]

TABLE S6 Factors and concerns related to cross-border work intention across latent empowerment profiles [n (%)]

| Item | Overall  (N=3671) | Profile 1  (N=464) | Profile 2  (N=2585) | Profile 3  (N=622) | χ² | *P* |
| --- | --- | --- | --- | --- | --- | --- |
| **Factors influencing the decision to work abroad** | | |  |  |  |  |
| Good salary | 3242(88.3) | 418(90.1) | 2279(88.2) | 545(87.6) | 1.759 | 0.415 |
| Good promotion opportunities | 2447(66.7) | 278(59.9) | 1732(67.0) | 437(70.3) | 13.259 | 0.001 |
| Relatives/friends in the area | 1157(31.5) | 177(38.1) | 789(30.5) | 191(30.7) | 10.822 | 0.004 |
| Probation period | 1525(41.6) | 204(44.1) | 1068(41.3) | 254(40.8) | 1.303 | 0.521 |
| Relocation allowance | 1965(53.5) | 274(59.1) | 1354(52.4) | 337(54.2) | 7.169 | 0.028 |
| Easy settlement for my family | 2309(62.9) | 303(65.3) | 1607(62.2) | 399(64.1) | 2.158 | 0.340 |
| **Factors of concern when considering cross-border work** | | | |  |  |  |
| Salary | 2985(81.3) | 404(87.1) | 2088(80.8) | 493(79.3) | 12.336 | 0.002 |
| Promotion opportunities | 2000(54.5) | 231(49.8) | 1413(54.7) | 356(57.2) | 6.063 | 0.048 |
| Cultural differences | 2133(58.1) | 266(57.3) | 1510(58.4) | 357(57.4) | 0.345 | 0.841 |
| Communication/language barriers | 2538(69.1) | 332(71.6) | 1786(69.1) | 420(67.5) | 2.029 | 0.363 |
| Differences in dietary habits | 1177(32.1) | 166(35.8) | 818(31.6) | 193(31.0) | 3.450 | 0.178 |
| Being away from family | 2435(66.3) | 324(69.8) | 1728(66.8) | 383(61.6) | 9.147 | 0.010 |
