# Supplementary material for: Nurses’ Cross‐Border Work Intentions Driven by Psychological Empowerment: A Cross‐Sectional Study
Source: J Nurs Manag. 2026 Mar 9;2026:8714790. doi: 10.1155/jonm/8714790 (PMC12968889; doi:10.1155/jonm/8714790)
Supplement: Supplementary file 7 — Supporting Information 7 TABLE S7: Binary logistic regression of cross‐border intention. [file JONM-2026-8714790-s004.docx]

TABLE S7 Binary logistic regression of cross-border intention

| Independent variables | B | SE | Wald χ value | OR (95% CI) | *P* |
| --- | --- | --- | --- | --- | --- |
| *Model 1: Core-Driven empowerment profile as reference group* | | | | | |
| Medium psychological empowerment | -0.880 | 0.132 | 44.219 | 0.415 (0.320, 0.538) | <0.001 |
| Medium-high psychological empowerment | -0.545 | 0.092 | 35.090 | 0.580 (0.484, 0.694) | <0.001 |
| *Model 2:* *Adaptive empowerment profile as reference group* | | | | | |
| Medium psychological empowerment | -0.433 | 0.109 | 15.896 | 0.648 (0.524, 0.802) | <0.001 |
| Note: Sex, age, education level, marital status, salary, job title, years of work experience, type of work organization, specialist nurse qualification, and work night shifts are covariates. Likelihood ratio: χ^2^ = 162.551, *P* < 0.001. -2 Log Likelihood = 4763.395, Cox and Snell R^2^ = 0.043, Nagelker’ke R^2^ = 0.059. Hosmer-Lemeshow goodness of fit test: χ^2^ = 3.237, *P* = 0.919. | | | | | |
| Abbreviations: SE, standard error; OR, odds ratio; CI, confidence interval. | | | | | |
