# Supplementary material for: Nurses’ Cross‐Border Work Intentions Driven by Psychological Empowerment: A Cross‐Sectional Study
Source: J Nurs Manag. 2026 Mar 9;2026:8714790. doi: 10.1155/jonm/8714790 (PMC12968889; doi:10.1155/jonm/8714790)
Supplement: Supplementary file 8 — Supporting Information 8 TABLE S8: Cross‐border intention (yes, no, undecided) across latent empowerment profiles. [file JONM-2026-8714790-s005.docx]

TABLE S8 Cross-border intention (Yes, No, Undecided) across latent empowerment profiles (*n* = 3,671)

| Independent variables | cross-border intention | | | | | | Non cross-border intention | | | | | |  |  |
| --- | --- | --- | --- | --- | --- | --- | --- | --- | --- | --- | --- | --- | --- | --- |
|  | Estimate | SE | t/Z^b^ | OR 95% CI | *P* | Estimate | | SE | t/Z^b^ | OR 95% CI | *P* |  |  |  |
| **Fixed Effects^a^** |  |  |  |  |  |  | |  |  |  |  |  |  |  |
| **Latent Profile (ref: Core-Driven Empowerment Profile as reference group)** | | | | | | | | | | | | | | |
| Constrained Empowerment Profile | -0.934 | 0.159 | -5.861 | 0.393(0.287,0.537) | <0.001 | -0.061 | | 0.167 | -0.367 | 0.941(0.678,1.305) | 0.714 |  |  |  |
| Adaptive Empowerment Profile | -0.670 | 0.116 | -5.783 | 0.512(0.408,0.642) | <0.001 | -0.222 | | 0.129 | -1.726 | 0.801(0.623,1.031) | 0.084 |  |  |  |
| **Random Effects** |  |  |  |  |  |  | |  |  |  |  |  |  |  |
| Random-intercept variance(τ2) | 0.015 | 0.014 | 1.098 | - | 0.272 | 0.023 | | 0.028 | 0.847 | - | 0.397 |  |  |  |
| ICC | 0.0045 |  |  |  |  | 0.0069 | |  |  |  |  |  |  |  |
| **Model Fit Indices** |  |  |  |  |  |  | |  |  |  |  |  |  |  |
| -2 Log Likelihood(-2LL) | 26879.738 | | | | | | | | | | | | |  |
| Corrected Akaike Information Criterion (AICc) | 26883.741 | | | | | | | | | | | | |  |
| Bayesian Information Criterion (BIC) | 26896.122 | | | | | | | | | | | | |  |

Note: Sex, age, education level, marital status, salary, job title, years of work experience, type of work organization, specialist nurse qualification, and work night shifts are covariates.

a. Undecided cross-border intention group was used as the reference group.

b. The t/Z column reports t-values for fixed effect coefficients and Z-values for the significance test of random intercept variances.

Abbreviations: ICC, intraclass correlation coefficient; SE, standard error; OR, odds ratio; CI, confidence interval.
