# Supplementary material for: Nurses’ Cross‐Border Work Intentions Driven by Psychological Empowerment: A Cross‐Sectional Study
Source: J Nurs Manag. 2026 Mar 9;2026:8714790. doi: 10.1155/jonm/8714790 (PMC12968889; doi:10.1155/jonm/8714790)
Supplement: Supplementary file 9 — Supporting Information 9 TABLE S9: Cross‐border intention by latent empowerment profiles (undecided group excluded). [file JONM-2026-8714790-s006.docx]

TABLE S9 Cross-border intention by latent empowerment profiles

(undecided group excluded, *n*=2581)

| Independent variables | Estimate | SE | t/Z^b^ | OR (95% CI) | *P* |
| --- | --- | --- | --- | --- | --- |
| ***Model 1: Core-Driven Empowerment group as reference group*** | | | | | |
| **Fixed Effects^a^** | | | | | |
| Constrained Empowerment | -0.867 | 0.157 | -5.509 | 0.420(0.308,0.572) | <0.001 |
| Adaptive Empowerment Profile | -0.448 | 0.1121 | -3.995 | 0.639(0.513,0.796) | <0.001 |
| Overall model test (F-test) | F=15.649, df₁=2, df₂=2551 | | | | <0.001 |
| **Random Effects** |  |  |  |  |  |
| Random-intercept variance(τ^2^) | 0.046 | 0.036 | 1.265 | - | 0.206 |
| ICC | 0.014 | | | | |
| **Model Fit Indices** |  | | | | |
| -2 Log Likelihood(-2LL) | 11246.535 | | | | |
| AICc | 11248.536 | | | | |
| BIC | 11254.379 | | | | |
| ***Model 2: Adaptive Empowerment group as reference group*** | | | | | |
| **Fixed Effects^a^** | | | | | |
| Constrained Empowerment | -0.427 | 0.129 | -3.289 | 0.653(0.506,0.842) | 0.001 |
| Overall model test (F-test) |  |  | F=10.815, df₁=1, df₂=2072 | | 0.001 |
| **Random effects** |  |  |  |  |  |
| Random-intercept variance(τ^2^) | 0.043 | 0.038 | 1.138 | - | 0.255 |
| ICC | 0.0129 |  |  |  |  |
| **Model Fit Indices** |  |  |  |  |  |
| -2 Log Likelihood(-2LL) | 9078.366 | | | |  |
| AICc | 9080.368 | | | |  |
| BIC | 9086.002 | | | |  |
| Note: This table is based on the final analytical sample of 2,581 respondents, after excluding 1,099 participants who selected the “undecided” option for cross-border work intention.  Sex, age, education level, marital status, salary, job title, years of work experience, type of work organization, specialist nurse qualification, and work night shifts are covariates.  a. Non-cross-border intention group was used as the reference group.  b. The t/Z column reports t-values for fixed effect coefficients and Z-values for the significance test of random intercept variances.  Abbreviations: ICC, Intraclass correlation coefficient; AICc, Corrected Akaike Information Criterion; BIC, Bayesian Information Criterion; SE, standard error; OR, odds ratio; CI, confidence interval. | | | | | |
